# A Rare Case of Acute Necrotizing Pancreatitis Infected With Prevotella Species

**DOI:** 10.7759/cureus.36145

**Published:** 2023-03-14

**Authors:** Cindy Traboulsi, Nikola Gligorijevic

**Affiliations:** 1 Department of Internal Medicine, University of Pittsburgh Medical Center, Pittsburgh, USA

**Keywords:** infection, prevotella, infected pancreatic necrosis, hypertriglyceridemia, acute pancreatitis

## Abstract

Acute pancreatitis is one of the leading causes of gastrointestinal-related hospitalizations in the United States. One of the complications of acute pancreatitis is infected pancreatic necrosis. We present a rare case of acute necrotizing pancreatitis infected with *Prevotella* species in a young patient. We demonstrate the importance of early suspicion of complicated acute pancreatitis and the need for early intervention to prevent hospital re-admission and improve the morbidity and mortality associated with infected pancreatic necrosis.

## Introduction

Acute pancreatitis is one of the leading causes of gastrointestinal-related hospitalizations in the United States [1]. Around 20% of patients with acute pancreatitis are characterized as severe and develop complications, including necrotizing pancreatitis [2]. *Enterococci* are the most commonly isolated species in infected pancreatic necrosis, followed by *Escherichia Coli*, with anaerobic bacteria being more rarely involved [3]. We present a rare case of acute necrotizing pancreatitis infected with *Prevotella* species in a young patient.

This article was previously presented as a meeting abstract at the 2022 ACG Annual Scientific Meeting on October 25, 2022.

## Case presentation

This is a case of a 28-year-old man with a past medical history of attention deficit hyperactivity disorder and hypertension who initially presented with acute epigastric pain radiating to the left. He was found to have a lipase level of 3897 U/L, acute interstitial pancreatitis with extensive peri-pancreatic inflammation on a computerized tomography (CT) scan, and a triglyceride level of 2716 mg/dL. He was admitted to the general medicine floor for pain control, intravenous fluids, and an insulin infusion. During his stay, he also received a therapeutic plasma exchange, given that his triglyceride levels remained persistently elevated, and was discharged nine days later upon the improvement of his symptoms.

He presented again 13 days later with worsening epigastric pain, an inability to tolerate oral intake, and an elevated white blood cell count. He was empirically started on intravenous (IV) antibiotics with cefepime 2g every eight hours and metronidazole 500mg every eight hours, in addition to a loading dose of vancomycin 2500mg followed by a dose of vancomycin 1500mg every 12 hours. On a CT scan, he was found to have a large pancreatic pseudocyst in addition to a probable small area of necrosis in the pancreatic body and head. He had a nasojejunal tube placed, and tube feedings were initiated. His antibiotics were stopped after two doses of each, as he was hemodynamically stable, and his imaging was not suggestive of infection. His symptoms gradually improved with pain control and antiemetics, and he was re-imaged before discharge 13 days later, with no changes seen on the CT scan.

**Figure 1:**
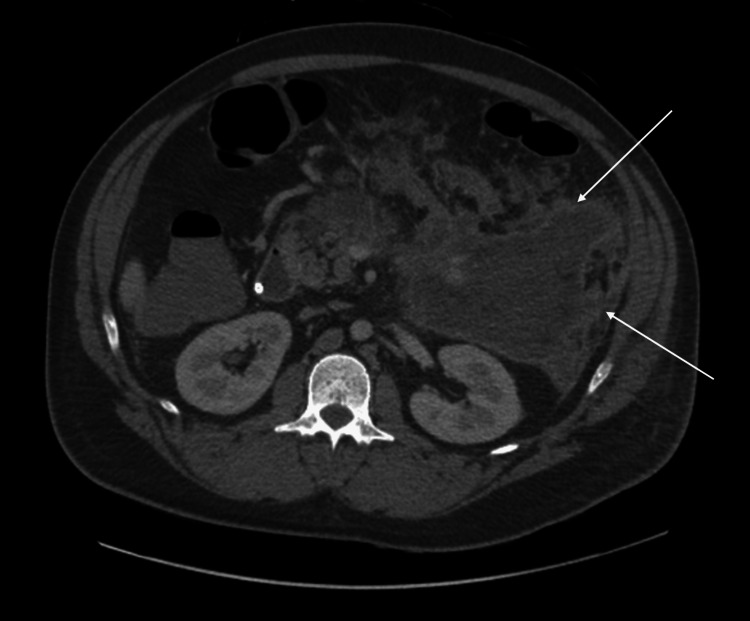
Necrotizing pancreatitis with large, evolving peri-pancreatic walled-off necrosis.

He presented again, six days post-discharge, with persistent abdominal pain. He had a CT scan that showed necrotizing pancreatitis with multi-loculated walled-off necrosis (Figure 1) in addition to pleural effusions. He had a new oxygen requirement of 2L and underwent a thoracentesis with an improvement in his respiratory symptoms. He was discharged home eight days later as he was clinically stable. 

**Figure 2:**
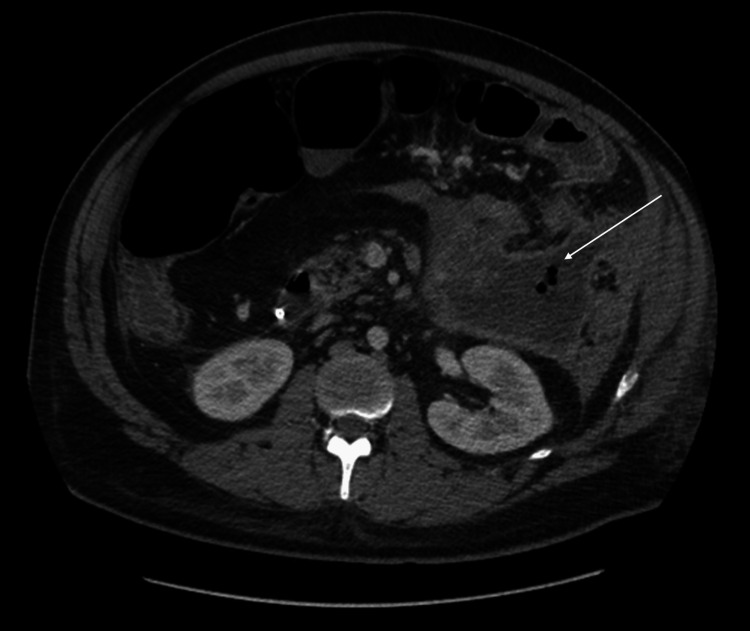
Large, evolving peri-pancreatic walled-off necrosis with evidence of new gastric locules within the collection.

He presented again one week after discharge with worsening abdominal pain and emesis. He was found to have pancreatic walled-off necrosis with gas, concerning superimposed infection (Figure 2), gastric outlet obstruction, and colonic obstruction, as seen on the CT scan. A percutaneous drain was placed. His course was complicated by sepsis, and he was started on broad-spectrum antibiotics (cefepime 2g IV every eight hours, metronidazole 500mg IV every eight hours, and vancomycin dosed per the pharmacokinetics service) after fluid collection. His cultures grew moderate amounts of *staphylococcus aureus* and *streptococcus intermedius* and heavy amounts of *prevotella buccae*, *prevotella denticola*, and *fusobacterium*. He was then narrowed down to piperacillin-tazobactam, 4.5 g IV every six hours. He had a repeat thoracentesis for recurrent bilateral effusions. His course was complicated by worsening abdominal pain and distention. A repeat CT revealed increasing colonic and small bowel dilatation due to a strictured descending colon, likely due to inflammation from the peripancreatic walled-off necrosis. A gastroview enema was also done and demonstrated a probable fistula tract. He eventually underwent percutaneous endoscopic gastrostomy tube placement and the creation of a loop transverse colostomy. Post-operatively, he was transitioned to oral amoxicillin-clavulanic acid (875/125mg twice daily) for an additional four-week course with planned outpatient follow-up.

## Discussion

We describe an atypical case of hypertriglyceridemia-induced pancreatitis complicated by a necrotizing infection with heavy Prevotella species. The most common causes of pancreatitis are gallstones, followed by alcohol, which account for at least 40 and 30 percent of cases, respectively [4]. Hypertriglyceridemia is the third most common cause of acute pancreatitis and, in general, accounts for around 5% of all cases [5]. Multiple studies have aimed to compare the severity and prognosis of acute pancreatitis based on the causing factor. A study from China demonstrated that the incidences of pancreatic necrosis, infected pancreatic necrosis, and organ failure were higher in patients with hypertriglyceridemia-induced pancreatitis as compared to acute pancreatitis from other causes (28.3% vs. 18.1%, 6.1% vs. 3.7%, and 35.8% vs. 29.1%, respectively) with p<0.01 [6]. Another study done in Spain by Navarro S et al. [7] showed that patients with hypertriglyceridemia-induced pancreatitis had more severe disease and more complications when compared to patients with gallstone pancreatitis [7]. Additionally, increasing levels of triglycerides were associated with a higher rate of complications, including pancreatic necrosis and organ failure [8,9].

Pancreatic necrosis is usually seen in around 20% of patients with acute pancreatitis [2]. About 33% of patients with necrotizing pancreatitis develop infected necrosis [10], which is a major cause of mortality in patients with acute pancreatitis. Different studies have attempted to identify the bacteria most commonly isolated in infected pancreatic necrosis. A study by Mowbray NG et al. [11] identified *Enterococcus faecalis* as the most frequently isolated microbe (22.5%), followed by both *Enterococcus faecium* (20%) and *Escherichia coli* (20%) [11]. Anaerobes were isolated in 12.5% of cases and were always associated with a polymicrobial culture [11]. A study by Ashley SW et al. [12] identified 99 patients with necrotizing pancreatitis, 34 of whom had infected necrosis. *Staphylococcal *species accounted for 33% of isolates, followed by *Escherichia coli* (22%) and *Klebsiella* (13%) [12]. Infections with anaerobic bacteria are rare, and *clostridium perfringens* are usually the primary organism involved [13]. *Prevotella* is a genus of gram-negative anaerobic rods that usually colonizes human mucosal surfaces, including the oral cavity, skin, and gastrointestinal tract [14]. The gut microbiome plays an important role in acute pancreatitis. Preserving the integrity of the gut microbiome during the acute phase is thought to reduce complications, including the translocation of intestinal bacteria that could lead to infected necrosis [15]. The involvement of *Prevotella* in gut dysbiosis has not been consistently described in the literature. Studies have shown that increased *Prevotella* in the gut is linked to obesity, hypertension, and non-alcoholic fatty liver disease [16]. However, other studies exhibited no association with obesity or diabetes [16]. To our knowledge, only one case report presents a patient with acute necrotizing pancreatitis infected with *Prevotella* species. The patient was 40 years old and had insulin-dependent diabetes in addition to a history of chronic alcoholism. His collection was drained, he underwent a necrosectomy, and he improved on antibiotics [17].

Our patient developed severe pancreatitis, was admitted on four different occasions to the hospital, and was infected with a rare anaerobe despite his young age and lack of significant comorbidities. There are currently no validated risk fact­­ors for developing infected pancreatic necrosis in patients with pancreatitis, but studies have suggested that patients with hypertriglyceridemia-induced pancreatitis, especially with higher triglyceride levels, might be at increased risk of developing the severe disease [5-7,9]. Our patient had very high triglyceride levels on presentation, which most likely increased his risk of complications. Identifying that he is at high risk early on could have potentially led to a closer follow-up, earlier initiation of antibiotics, and potentially early drainage of his collection, hopefully preventing compression, stricturing, and bowel obstruction. Prevotella growth on his anaerobic culture might have been related to his obesity and hypertension, although more studies need to be done to verify this association.

## Conclusions

Acute necrotizing pancreatitis, especially if infected, is associated with poor outcomes. We highlight a rare case of hypertriglyceridemia-induced pancreatitis complicated by necrotizing infection with heavy *Prevotella* species. We demonstrate the importance of early suspicion of complicated acute pancreatitis and the need for early intervention to prevent hospital re-admission and improve the morbidity and mortality associated with infected pancreatic necrosis.
